# GmARF15 Enhances the Resistance of Soybean to *Phytophthora sojae* by Promoting *GmPT10d* Expression in Response to Salicylic Acid Signalling

**DOI:** 10.3390/ijms26010191

**Published:** 2024-12-29

**Authors:** Yuhan Huo, Haiyuan Chen, Zhuo Zhang, Yang Song, Siyan Liu, Piwu Wang, Sujie Fan

**Affiliations:** Plant Biotechnology Center, College of Agronomy, Jilin Agriculture University, Changchun 130118, China; huoyuhan666@163.com (Y.H.);

**Keywords:** soybean, *Phytophthora sojae*, GmARF15, *GmPT10d*, salicylic acid

## Abstract

Phytophthora root and stem rot caused by *Phytophthora sojae* (*P. sojae*) is a globally prevalent oomycete disease. The use of resistant cultivars is an effective and environmentally friendly strategy to manage this disease. It is important to understand the molecular mechanisms underlying the response of *Glycine max* (soybean) to *P. sojae* infection. In this study, we demonstrated that an isoflavonoid-specific prenyltransferase gene (*GmPT10d*, Glyma.10G070300) was significantly upregulated in the soybean cultivar Williams 82 with high resistance to *P. sojae* infection. Transgenic soybean seedlings overexpressing *GmPT10d* exhibited enhanced resistance to *P. sojae*, and those subjected to RNA interference showed increased susceptibility to the pathogen. Yeast-one-hybrid and electrophoretic mobility shift assays revealed that GmARF15 could directly bind to the promoter of *GmPT10d*. Further analysis of the *GmARF15* function showed that transgenic soybean seedlings overexpressing *GmARF15* also exhibited enhanced resistance to *P. sojae*. Transactivation assay, luciferase assay, and qPCR analysis showed that GmARF15 could promote the expression of *GmPT10d*. Further analysis indicated that elevated salicylic acid levels were associated with increased expression of *GmARF15* and *GmPT10d*. Taken together, these findings reveal a regulatory mechanism by which GmARF15 enhances soybean resistance to *P*. *sojae*, potentially by promoting the expression of *GmPT10d* through the salicylic acid signaling pathway.

## 1. Introduction

Soybean (*Glycine max*) is a major source of plant-based protein and oil, and plays an important role in global agricultural production. A major factor causing the decline in soybean yields is the prevalence of various pathogens [1,2]. Phytophthora root and stem rot caused by the oomycete pathogen *Phytophthora sojae* (*P. sojae*) is responsible for annual yield losses valued at approximately USD 200 million in the United States and USD 1–2 billion worldwide [3]. At present, chemical control of phytophthora root and stem rot is challenging; therefore, the use of resistant cultivars represents an effective and environmentally friendly strategy to manage this disease [4,5]. In breeding cultivars, it is important to understand the molecular mechanisms by which soybean plants respond to *P. sojae* infection.

Phytoalexins play a vital role in plant disease resistance. Glyceollins are pterocarpan phytoalexins derived from isoflavonoids in soybean plants and are synthesized via the isoflavonoid branch of the phenylpropanoid pathway [6,7,8,9]. The expression of many isoflavonoid biosynthetic genes is induced by pathogenic bacteria to enhance disease resistance [10,11]. Soybean produces multiple forms of glyceollins through various prenylation reactions catalyzed by prenyltransferases (PTs) acting on either the C-2 or C-4 of a pterocarpan glycinol [7,9,12]. Researchers have identified 11 isoflavonoid-PT-encoding genes in soybean and found that the expression of five of these genes is upregulated in response to *P. sojae* infection [13]. *GmPT01* is possibly involved in the partial resistance to root rot disease and can be utilized in breeding resistant cultivars [13]. Therefore, it is crucial to elucidate the molecular mechanisms by which PT-encoding genes contribute to resistance against *P. sojae* infection.

Auxin response factor genes (*ARFs*) are widely present in plants and play important roles in plant development and stress responses such as seed germination, flower development, fruit maturation, leaf senescence, and responses to biotic and abiotic stresses [14,15,16,17,18]. Under salt stress, the expression levels of most *SlARF* genes were downregulated, while some of these genes, such as *SlARF1*, *SlARF4*, and *SlARF19*, were significantly upregulated [19]. Most *BvARF* genes in sugar beet showed varying degrees of differential expression in response to salt stress [20]. Rice OsARF17 played a key role in defense against various plant viruses such as *Fijiviruses*, *Tenuivirus*, and *Cytorhabdovirus*, with the proteins of these viruses interacting with OsARF17 through distinct mechanisms [21]. In potato, StARF10, a target of miR160, was shown to bind to the promoter of *StNPR1* and regulate its expression in response to infection by necrotrophic pathogen [22]. ShARF04, ShARF07, and ShARF17 functioned as positive regulators during sugarcane infections by *Acidovorax avenae* subsp. *avenae* and *Xanthomonas albilineans* [23]. However, there is no report on the research of the *ARF* genes in soybean response to *P. sojae*.

Salicylic acid (SA) is an important plant hormone that regulates the signaling pathways associated with plant disease resistance [24,25]. Moreover, SA plays crucial roles in basal defense and the amplification of local immune responses, as well as in the establishment of systemic acquired resistance [26]. Quercetin induces pathogen resistance in *Arabidopsis* by promoting SA biosynthesis [27]. Systemic acquired resistance in *Arabidopsis* can be triggered by direct pathogen infection or SA treatment [28]. SA activates BIN2 phosphorylation of TGA3, which in turn promotes *Arabidopsis PR* gene expression and disease resistance [29]. Quercetin-induced SA accumulation triggers the expression of *PR* genes through the activation of NPR1 [30]. Phenylalanine ammonia lyase-mediated SA biosynthesis contributes to nematode resistance in wheat [31]. To sum up, the role of the ARF combined with *PTs* module in mediating the response to *P. sojae* infection in the presence of exogenous SA remains unclear.

In a previous study, RT-PCR analysis demonstrated that a PT-encoding gene *GmPT10d* (Glyma.10G070300) was significantly up-regulated in the soybean cultivar L76-1988 highly resistant to *P. sojae* infection [13]. However, the role of this gene in the accumulation of intracellular ions in different genetic backgrounds remains to be characterized. In this study, *GmPT10d* was isolated from the resistant soybean cultivar Williams 82. Transgenic soybean seedlings overexpressing *GmPT10d* exhibited enhanced resistance to *P. sojae*, whereas RNA interference resulted in increased susceptibility to this pathogen. We identified the transcription factor GmARF15 as an upstream regulator of *GmPT10d*, which positively regulated the resistance against *P. sojae*. Further analysis indicated that GmARF15 could promote the expression of *GmPT10d*. In others, SA accumulation resulted in elevated *GmARF15* and *GmPT10d* expression. Therefore, we proposed a model illustrating the role of GmARF15-*GmPT10d* in soybean defense against *P. sojae* infection.

## 2. Results

### 2.1. Expression Pattern and Sequence Characteristics of GmPT10d

**Figure 1:**
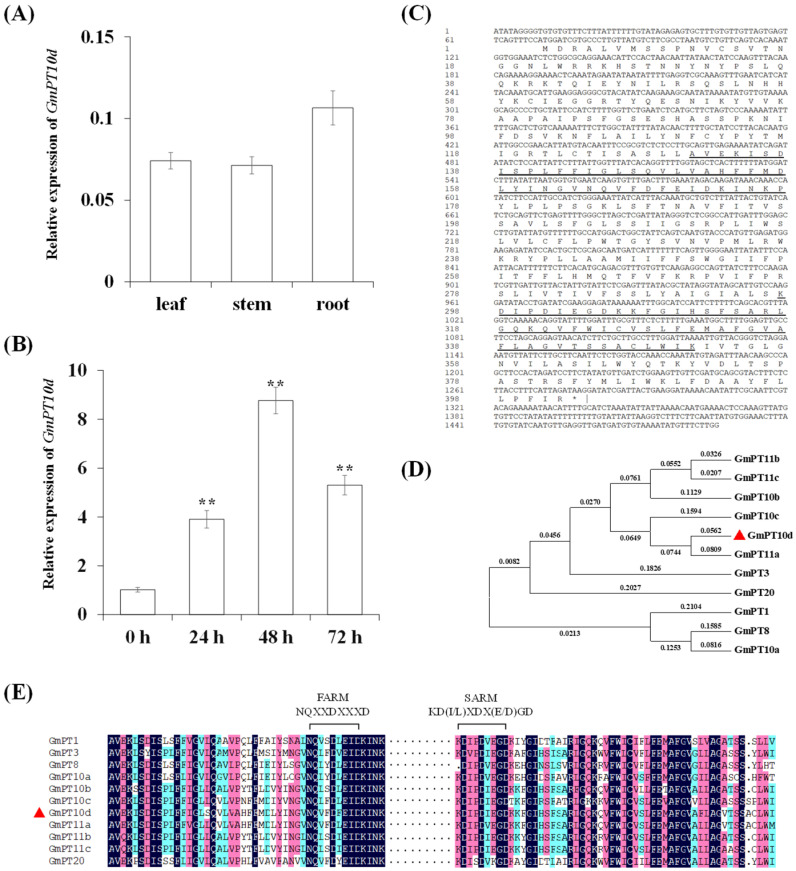
Expression pattern and sequence characteristics of *GmPT10d*. (**A**) *GmPT10d* mRNA levels in various soybean tissues. (**B**) *GmPT10d* mRNA levels in soybean roots infected with *P*. *sojae*. Soybean root samples were collected at 0 (control), 24, 48, and 72 h post-inoculation. Three biological replicates were used for each sample and Student’s *t*-test (** *p* < 0.01) was performed to determine the statistical significance of differences. Error bars represent ±SD. (**C**) The *GmPT10d* gene sequence and the GmPT10d protein sequence. The first aspartate-rich motif (FARM) and the second aspartate-rich motif (SARM) are underlined. (**D**) Phylogenetic relationships between GmPT10d and other soybean PT family members. (**E**) Multiple sequence alignment of the aspartate-rich motifs using DNAMAN 6.0 software. Abridged version of alignment is shown to highlight both first and second aspartate-rich motifs. Red triangles is GmPT10d.

To study *GmPT10d* expression, we first examined the accumulation of *GmPT10d* in various tissues of the soybean seedlings by qPCR. *GmPT10d* was constitutively expressed primarily in the roots, followed by the leaves and the stems (Figure 1A). We further explored the expression pattern of *GmPT10d* during *P. sojae* infection. As shown in Figure 1B, the expression of *GmPT10d* was significantly upregulated in the highly resistant soybean cultivar Williams 82 after *P. sojae* infection (*p* < 0.01), and the accumulation of *GmPT10d* peaked at 48 h post-inoculation. The results suggested that *GmPT10d* may be involved in the response of soybean to *P. sojae* infection.

The complete sequence of *GmPT10d* was cloned from Williams 82 using the RT-PCR technique, which contained a 1209-bp open reading frame that encodes a 402-amino acid protein with the first aspartate-rich motif (FARM) and the second aspartate-rich motif (SARM) (Figure 1C). Soybean PT family contains 11 members [13]. Phylogenetic analysis showed that the 11 soybean PTs were conserved and GmPT10d clustered in the same branch as GmPT11a (Figure 1D). Multiple sequence alignment of soybean PTs clearly highlighted the presence of both FARM and SARM (Figure 1E).

### 2.2. GmPT10d Enhances Resistance to P. sojae in Soybean Seedlings

**Figure 2:**
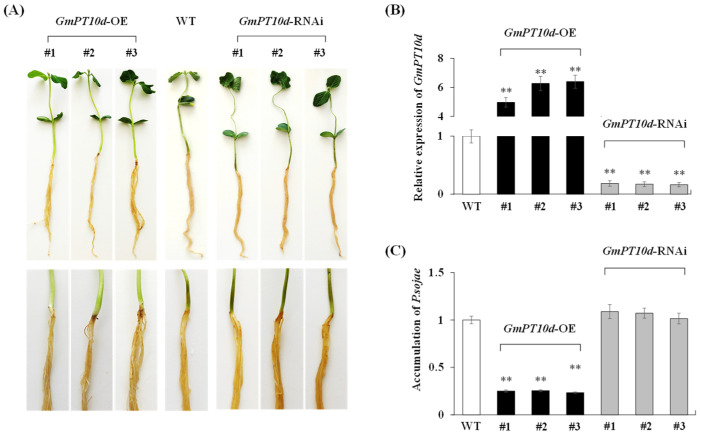
Disease symptoms of the transgenic and WT soybean seedlings in the soybean root hydroponic assay. (**A**) Phenotypes of WT, *GmPT10d*-OE transgenic, and *GmPT10d*-RNAi soybean seedlings treated for 48 h with zoospores of *P. sojae* in the soybean root hydroponic assay. (**B**) The relative expression levels of *GmPT10d* in WT, *GmPT10d*-OE transgenic, and *GmPT10d*-RNAi soybean seedlings at 48 h post-inoculation. (**C**) Accumulation of *P. sojae* in WT, *GmPT10d*-OE transgenic and *GmPT10d*-RNAi soybean seedlings at 48 h post-inoculation. WT, wild type. WT soybean seedlings were used as control. Three biological replicates were used for each sample and Student’s *t*-test (** *p* < 0.01) was used to determine statistical significance. Error bars represent ±SD.

In the current study, we further explored the network through which *GmPT10d* regulated the response to *P. sojae* infection. Specifically, we constructed a plant overexpression vector 35S::*GmPT10d* and an RNA interference vector 35S::*GmPT10d*-RNAi. Both vectors were subsequently transformed into soybean seedlings using an *Agrobacterium*-mediated transformation system. In T2 generation, we tested three positive soybean seedlings from each positive transgenic plant of T0 generation on the LibertyLink strip detection of BAR or PAT protein (Figures S1 and S2). We evaluated the resistance to *P. sojae* in wild type (WT) and transgenic (*GmPT10d*-OE and *GmPT10d*-RNAi) soybean seedlings via incubation with *P*. *sojae* zoospores in a root hydroponic assay. At 48 h post-inoculation, a remarkable difference in disease symptom development was observed. The *GmPT10d*-OE transgenic soybean seedlings remained firm, with only slight browning of the roots, while the WT and *GmPT10d*-RNAi soybean seedlings exhibited extended lesions, with the entire plant wilting, turning brown, and emitting a foul odor (Figure 2A). At 48 h post-inoculation, the *GmPT10d*-OE transgenic soybean seedlings showed a higher rate of increase in *GmPT10d* expression compared with WT soybean seedlings, and the *GmPT10d*-RNAi soybean seedlings showed a lower rate of increase in *GmPT10d* expression compared with WT soybean seedlings (Figure 2B). Moreover, the relative accumulation of *P. sojae* was significantly lower (*p* < 0.01) in the *GmPT10d*-OE transgenic soybean seedlings compared with WT and *GmPT10d*-RNAi soybean seedlings at 48 h post-inoculation (Figure 2C). These results demonstrated that *GmPT10d* acts as a positive regulator of soybean resistance to *P. sojae* infection.

### 2.3. Identification of Upstream Transcription Factors Regulating GmPT10d

**Figure 3:**
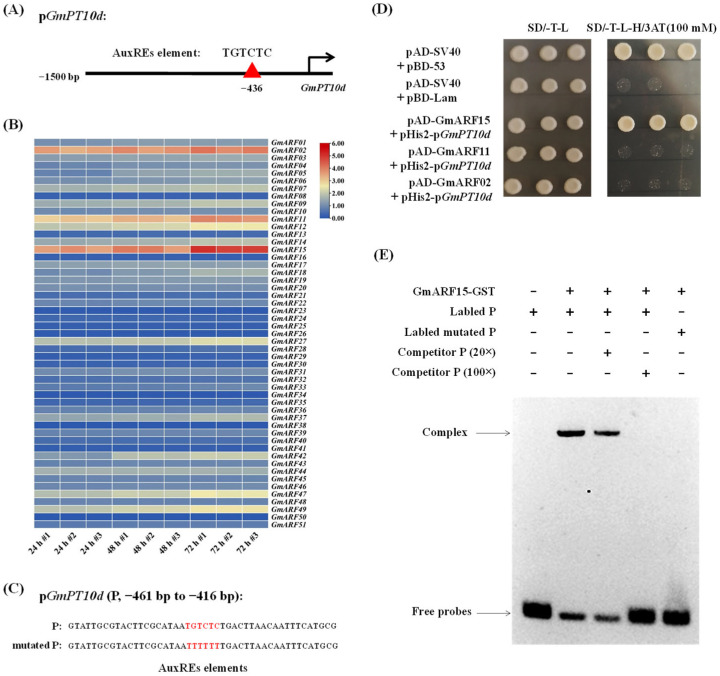
Identification of upstream transcription factors of *GmPT10d*. (**A**) The auxin-response elements of *GmPT10d* promoter. (**B**) Heat map of the expression patterns of 51 soybean *ARF* genes in response to *P. sojae*, as determined by qPCR analysis. The samples were collected at 24, 48, and 72 h post-inoculation. Three biological replicates were used for each sample. (**C**) Oligonucleotide of the P or mutated P from *GmPT10d* promoter. Red fonts indicate AuxREs elements. (**D**) Y1H assay of GmARF2, GmARF11, and GmARF15 proteins binding to the P site that contains the TGTCTC from *GmPT10d* promoter. (**E**) Electrophoretic mobility shift assay of GmARF15 protein binding to the P or mutated P probe. Arrows indicate complex DNA and free probes, respectively.

To identify the upstream genes that regulate the expression of *GmPT10d*, we analyzed the 1.5 kb promoter sequence of *GmPT10d* and discovered a TGTCTC binding site for the ARF transcription factor located in the −436 to −441 region (Figure 3A). Subsequently, we analyzed the expression patterns of 51 members of the soybean *ARF* gene family in response to *P. sojae*. The results of qPCR showed that the expression of *GmARF2*, *GmARF11*, and *GmARF15* was significantly upregulated at 24, 48, and 72 h post-inoculation, and the expression levels increased with increasing duration of infection (Figure 3B). The partial melting curves of the qPCR are listed in Figure S3. A Y1H assay was performed to assess the direct binding of GmARF2, GmARF11, and GmARF15 proteins to the *GmPT10d* promoter. The assay focused on the −461 to −416 region (P) of the *GmPT10d* promoter (Figure 3C). The results of Y1H showed that the positive control (pAD-SV40 + pBD-53) and yeast co-transformates (pAD-GmARF15 + pHis2-P) could grow normally on SD/-Trp-Leu-His with 100 mM 3-aminotriazole, whereas yeast co-transformates (pAD-GmARF02 + pHis2-P or pAD-GmARF11 + pHis2-P) and the negative control (pAD-SV40 + pBD-Lam) did not grow in the same medium. These results demonstrated that GmARF15 protein could directly bind to the −461 to −416 region of the *GmPT10d* promoter (Figure 3D). To further confirm that GmARF15 directly binds to the P region of the *GmPT10d* promoter in vivo, we conducted EMSA using purified GST-GmARF15 protein and the DNA fragments corresponding to the P region. As shown in Figure 3E, the recombinant GmARF15 protein specifically bound to the labeled P region but not to the labeled mutated P region. Increasing molar excesses of unlabeled P fragment (competitor) inhibited the binding. These findings indicated that GmARF15 specifically binds to the −461 to −416 region of the *GmPT10d* promoter in vitro.

### 2.4. Identification of the Transcriptional Activator GmARF15

**Figure 4:**
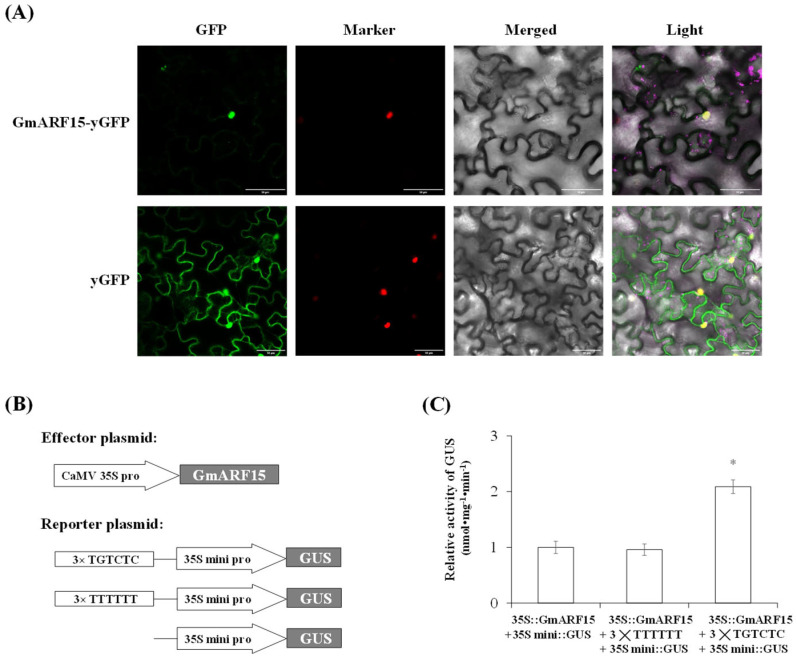
Subcellular localization and transactivation assay of GmARF15 protein. (**A**) Subcellular localization of GmARF15 protein in tobacco epidermal cells. Scale bars represent 20 μm. (**B**) Schematic diagram of effector plasmid and reporter plasmid constructs. (**C**) GUS activity analysis. The effector plasmid and reporter plasmid were co-transfected into Arabidopsis protoplasts. Relative GUS activity driven by 35S::*GmARF15* and 35Smini::*GUS* was used as a control. Three biological replicates were used for each sample and Student’s *t*-test (* *p* < 0.05) was used to determine statistical significance. Error bars represent ±SD.

To investigate the subcellular localization of GmARF15, the 35S::*GmARF15*-GFP (green fluorescence protein) was transformed into the leaf epidermal cells of tobacco (*Nicotiana tabacum*). The fluorescent signal of GmARF15-GFP fusion protein was exclusively localized to the nucleus, whereas the fluorescent signal of the control was in the cytoplasm and nucleus (Figure 4A). The transactivation assay was also performed in Arabidopsis protoplasts. The effector plasmid contains the recombinant 35S::*GmARF15*, and the reporter plasmid contains the 35S mini::*GUS* or a the recombinant 35S mini::*GUS* with three tandem copies of the TGTCTC or TTTTTT sequences (Figure 4B). The relative GUS activity driven by 35S::*GmARF15* and 35S mini::*GUS* with three tandem copies of the TGTCTC was approximately 2.0 times higher than that of the control (35S::*GmARF15* and 35Smini::*GUS* or 35S mini::*GUS* with three tandem copies of TTTTTT) (Figure 4C). These results suggest that GmARF15 is a TGTCTC sequence binding protein and functions as a transcriptional activator.

### 2.5. Overexpression of GmARF15 Enhances Soybean Resistance to P. sojae

**Figure 5:**
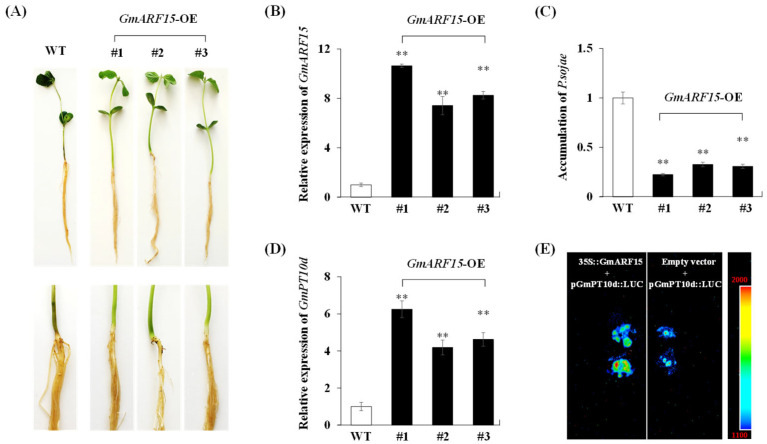
*GmARF15* overexpression enhances soybean resistance to *P. sojae*. (**A**) Phenotypes of WT and *GmARF15*-OE transgenic soybean seedlings treated with zoospores of *P. sojae* for 48 h in the soybean root hydroponic assay. (**B**) The relative expression levels of *GmARF15* in WT and *GmARF15*-OE transgenic soybean seedlings at 48 h post-inoculation. (**C**) Accumulation of *P. sojae* in WT and *GmARF15*-OE transgenic soybean seedlings at 48 h post-inoculation. (**D**) The relative expression levels of *GmPT10d* in WT and *GmARF15*-OE transgenic soybean seedlings at 48 h post-inoculation. WT, wild type. WT soybean seedlings were used as control. Three biological replicates were used for each sample and Student’s *t*-test (** *p* < 0.01) was used to determine statistical significance. Error bars represent ±SD. (**E**) Effect of GmARF15 on luciferase activation in tobacco leaf cells. Tobacco leaf cells transformed with p*GmPT10d*::*LUC* was used as control. Luciferase assay was performed at 3 days post-inoculation.

To further determine the potential role of *GmARF15* in response to *P. sojae* infection, transgenic soybean seedlings overexpressing *GmARF15* were generated. In T2 generation, we tested three positive soybean seedlings from each positive transgenic plant of T0 generation on the LibertyLink strip detection of BAR (Figure S4). We assessed the resistance of WT and transgenic (*GmARF15*-OE) soybean seedlings to *P. sojae* by incubation with *P. sojae* zoospores in a root hydroponic assay. At 48 h post-inoculation, the *GmARF15*-OE transgenic soybean seedlings exhibited much better phenotypes with firm and strong roots, whereas the WT soybean seedlings became wilted, soft, and showed extended lesions (Figure 5A). qPCR analysis revealed that the *GmARF15*-OE transgenic soybean seedlings showed a higher rate of increase in *GmARF15* expression compared with WT soybean seedlings after 48 h of *P. sojae* infection (Figure 5B). At 48 h post-inoculation, the relative accumulation of *P. sojae* was significantly (*p* < 0.01) lower in *GmARF15*-OE transgenic soybean seedlings than in WT soybean seedlings (Figure 5C). These results demonstrate that *GmARF15* is a positive regulator in soybean resistance to *P. sojae* infection.

Moreover, the expression of *GmPT10d* was significantly upregulated in *GmARF15*-OE transgenic soybean seedlings at 48 h post-inoculation (*p* < 0.01) (Figure 5D). To clarify the mechanism by which GmARF15 regulates the expression of *GmPT10d*, we performed a dual effector–reporter assay, using *GmARF15* under the control of the 35S promoter (35S::*GmARF15*) as the effector and the luciferase gene (*LUC*) controlled by 1.5 kb of the *GmPT10d* promoter (p*GmPT10d*) as the reporter. Then, the recombinant vectors 35S::*GmARF15* and p*GmPT10d*::*LUC* were transformed into tobacco leaves. As shown in Figure 5E, compared with the control containing only p*GmPT10d*::*LUC*, the tobacco leaves co-transformed with 35S::*GmARF15* and p*GmPT10d*::*LUC* showed increased LUC activation. Taken together, these results support that GmARF15 enhanced the resistance to *P. sojae* infection by increasing the expression of *GmPT10d*.

### 2.6. GmARF15 Improves Disease Resistance via SA Signaling

**Figure 6:**
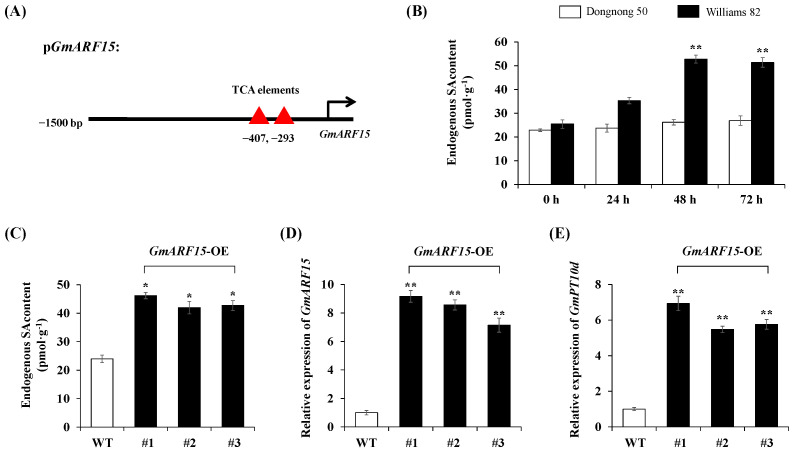
qPCR analysis of SA signaling in GmARF15-*GmPT10d* module. (**A**) Acting elements involved in SA responsiveness of *GmPT10d* promoter. (**B**) The endogenous SA content in *P. sojae*-resistant soybean cultivar Williams 82 and *P. sojae*-sensitive soybean cultivar Dongnong50 at 24, 48, and 72 h post-inoculation. (**C**) The endogenous SA content in both WT and *GmARF15*-OE transgenic soybean seedlings at 48 h post-inoculation. (**D**) The expression levels of *GmARF15* in WT and *GmARF15*-OE transgenic soybean seedlings after 48 h under SA treatment. (**E**) The expression levels of *GmPT10d* in WT soybean seedlings and *GmARF15*-OE transgenic soybean seedlings after 48 h of SA treatment. WT, wild type. Three biological replicates were used for each sample and Student’s *t*-test (* *p* < 0.05, ** *p* < 0.01) was used to determine statistical significance. Error bars represent ±SD.

SA plays an important role in plant immune response. We analyzed the 1.5 kb promoter sequence of *GmARF15* and discovered acting elements involved in SA responsiveness (Table 1, Figure 6A). Additionally, the endogenous SA content in *P. sojae*-resistant soybean cultivar Williams 82 was relatively high at 24, 48, and 72 h post-inoculation, and the content maximized at 48 h. In contrast, endogenous SA content remained at low levels in *P. sojae*-sensitive soybean cultivar Dongnong50, and the content showed no obvious changes during the treatment period (Figure 6B). To explore whether the GmARF15-*GmPT10d* module plays an important role in response to *P. sojae* infection, we measured the levels of endogenous SA content in both WT and *GmARF15*-OE transgenic soybean seedlings at 48 h post-inoculation. The results showed that the endogenous SA content was significantly (*p* < 0.05) higher in *GmARF15*-OE transgenic soybean seedlings than in WT soybean seedlings (Figure 6C). Then, we measured the expression levels of *GmARF15* and *GmPT10d* in WT and *GmARF15*-OE transgenic soybean seedlings after 48 h of SA treatment. The result showed that the expression levels of *GmARF15* and *GmPT10d* in *GmARF15*-OE transgenic soybean seedlings were significantly (*p* < 0.01) higher than those in WT soybean seedlings after SA treatment (Figure 6D,E). The above results indicate that GmARF15 promotes *GmPT10d* expression in response to SA signaling.

## 3. Discussion

Many isoflavonoid biosynthetic genes are involved in the regulation of pathogen attack. Powdery mildew infection induces differential foliar accumulation of isoflavonoids in the highly resistant *Medicago truncatula* genotype but not in the moderately susceptible genotype [32]. CaMYB39 positively modulates isoflavonoid accumulation and trichome density and confers defense against severe AB disease [33]. In soybean, 24 *CYP82* subfamily genes, which are involved in the flavonoid biosynthetic pathway, were differentially expressed in *P. sojae*-infected soybean varieties [34]. A previous study reported that five isoflavonoid-specific genes in soybean (*GmPT01*, *GmPT10a*, *GmPT10d*, *GmPT11a*, and *GmPT20*) showed induced expression in response to *P. sojae* Race 7 infection [13]. In this study, we further verified the role of *GmPT10d* in response to *P. sojae* Race 1 infection in soybean; the *GmPT10d*-OE transgenic soybean seedlings showed higher resistance and significantly lower *P. sojae* biomass compared with WT soybean seedlings (Figure 2), indicating that *GmPT10d* is a positive regulator in soybean resistance to *P. sojae* infection.

To construct a regulatory network for *GmPT10d* expression in response to *P. sojae* infection, we identified the transcription factor GmARF15 upstream of *GmPT10d*, which positively regulates resistance against *P. sojae* and promotes the expression of *GmPT10d* (Figure 3, Figure 4 and Figure 5). These results are consistent with reports showing that ARF transcription factors are involved in the regulation of pathogen infection networks. The *Pseudomonas syringae* type III effector AvrRpt2 promotes pathogen virulence via stimulating *Arabidopsis* auxin/indole-3-acetic acid protein turnover, making plants susceptible to pathogen invasion [35]. Rice plants overexpressing *OsARF17* were less susceptible to viral infection [21]. Conditional expression of the *ARF10* gene in *Brassica juncea* enhanced abscisic acid sensitivity, leading to fertile plants with increased tolerance to *Alternaria brassicicola* [16]. Other studies have shown that ARF transcription factors can specifically bind to the TGTC core sequence, with a preference for TGTCNN elements, and consequently regulate downstream gene expression, which is also consistent with our findings. ARFs can activate transcription of TGTCTC auxin-response elements (AuxREs) without directly binding to their DNA target sites, as demonstrated in transient expression assays employing auxin-responsive *GUS* reporter genes [36]. ARF1 forms a dimer and binds to palindromic TGTCTC AuxREs in Arabidopsis [37]. ARFs specifically bind to TGTCNN AuxREs, where they regulate auxin gene expression by either activating or repressing transcription [38]. Strawberry FveARF2 protein can directly bind to the TGTCTC element in the *FaSUT1*, *FaOMT*, and *FaCHS* promoters in vitro and in vivo [39]. In this study, we proved that GmARF15 binds to the TGTCTC sequence and functions as a transcriptional activator.

SA plays a key role in plant response to pathogen infection [40,41,42,43,44,45,46]. SA negatively regulates the expression of ShARF07 and ShARF17 in response to *Acidovorax avenae* subsp. *avenae* and *Xanthomonas albilineans* infections in sugarcane [23]. Exogenous SA treatment enhances apple resistance to *Penicillium expansum* [47]. Additionally, exogenous application of SA can also improve apple resistance to grey mold (*Botrytis cinerea*) [48]. In this study, we found that the endogenous SA content was significantly higher in *GmARF15*-OE transgenic soybean seedlings than in WT soybean seedlings at 48 h post-inoculation, and exogenous SA treatment increased *GmARF15* and *GmPT10d* expression levels (Figure 6). We therefore propose that GmARF15 enhances the resistance of soybean to *P. sojae* by promoting *GmPT10d* expression in response to SA signaling. In the follow-up work, it is valuable to explore glyceollin levels in transgenic and WT soybean seedlings after *P. sojae* infection, which will improve the interpretation of the findings that the GmARF15-GmPT10d module promotes the synthesis of glyceollin and enhances the resistance of soybean.

## 4. Conclusions

**Figure 7:**
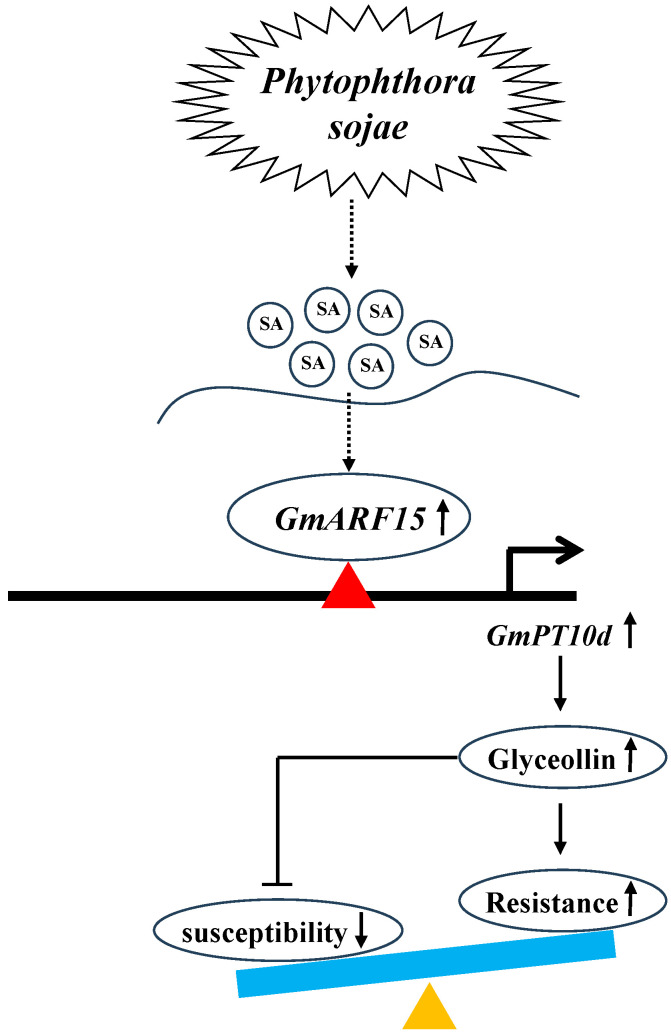
Proposed model of GmARF15-*GmPT10d* module in response to *P. sojae* infection in soybean.

We propose a model in which GmARF15-*GmPT10d* regulates soybean resistance to *P. sojae* (Figure 7). Upon infection by *P*. *sojae*, the concentration of SA increases, leading to upregulation of *GmARF15* expression; GmARF15 directly activates the expression of *GmPT10d*, which in turn enhances isoflavonoid biosynthesis, ultimately leading to increased resistance to *P. sojae* in soybean.

## 5. Materials and Methods

### 5.1. Plant Materials and Growth Conditions

Williams 82 is a soybean cultivar resistant to *P. sojae* infection, whereas Dongnong 50 is a soybean cultivar susceptible to *P. sojae* infection. In this study, Williams 82 was used for gene isolation, and Dongnong 50 was used for soybean transformation. Soybean seedlings were grown in pots filled with sterile vermiculite. The growth conditions were 14 h photoperiod, day/night temperatures of 25 °C/18 °C and relative humidity of 70 ± 10%. Soybean seedlings at the first-node stage (V1) [49] were used for various treatments. *Nicotiana benthamiana* was used for subcellular localization analysis and transient LUC transformation.

### 5.2. Oomycete Strains and Infection

The *P. sojae* race 1 (PSR01), which is the dominant race in Jilin Province, China, was generously provided by Professor Zhang Shuzhen at the Key Laboratory of Soybean Biology of the Chinese Education Ministry at Northeast Agricultural University. PSR01 was cultured on V8 juice agar in a polystyrene dish and activated by incubation for 7 days at 22–25 °C at the Plant Biotechnology Center, Jilin Agriculture University.

For disease resistance analysis, the roots of 14-day-old soybean seedlings were inoculated with *P*. *sojae* zoospores in a hydroponic assay. The inoculated seedlings were grown in a mist chamber at 25 °C with 90% relative humidity, a 14 h photoperiod, and a light intensity of 350 mol m^−2^ s^−1^. Soybean seedling roots were collected at 24, 48, and 72 h post-inoculation. Zoospore production followed the methods of Shrestha et al. (2016) [50] and Yang et al. (2021) [51].

### 5.3. RNA Extraction and qPCR Analysis

Total RNA was extracted from soybean roots using Trizol reagent (Invitrogen, Shanghai, China). qPCR was performed using One Step RT-PCR Kit (Code No. PCR-311, TOYOBO, Tokyo, Japan) on a QuantStudio 3 instrument (Thermo Fisher Scientific, Waltham, MA, USA). RNA quality was determined by 1.0% agarose gel electrophoresis. The primers for qPCR were designed using Primer 5 with an amplicon size range of 100–200 bp. SYBR green PCR protocol consisted of 95 °C for 1 min, followed by 40 cycles of 95 °C for 15 s, 60 °C for 15 s, and 72 °C for 45 s. The amplification product was validated using melting curve analysis at 1 °C intervals from 95 °C to 60 °C. Relative gene expression was analyzed using the 2^−ΔΔCT^ method with the conserved *GmActin* gene (Glyma.18G290800.1) as internal controls. All assays were carried out following the manufacturers’ protocols.

### 5.4. Genetic Transformation

To generate the 35S::*GmPT10d* and 35S::*GmARF15* overexpression constructs, their CDS sequences were amplified and inserted into the pCAMBIA3301 vector with the bar gene as a selective marker positioned between the *Nco* I and *Spe* I restriction sites. To generate the 35S::*GmPT10d*-RNAi silencing construct, a 387 bp fragment of *GmPT10d* was amplified and inserted into the pJawoh18 vector to create an inverted-repeat construct with the *PAT* gene as the selective marker. Then, the above recombinant vectors were transferred into *Agrobacterium tumefaciens* EHA105 via tri-parental mating. For soybean transformation, the cotyledonary nodes were used as explants according to the *Agrobacterium*-mediated transformation method described by Paz et al. (2004) [52] and Li et al. (2017) [53]. T2 transgenic soybean seedlings were verified by PAT/Bar LibertyLink strip (Envirologix, Portland, OR, USA), qPCR, and phenotypic analysis.

### 5.5. Assessment of Disease Responses in Soybean Seedlings

To examine the phenotype of soybean seedlings in response to *P. sojae* infection, roots of 14-day-old seedlings were inoculated with *P. sojae* zoospore suspension (approximately 10^4^ zoospores mL^− 1^) in a hydroponic assay. Inoculated seedlings were grown in a chamber at Jilin Agriculture University with a 14 h photoperiod, (a light intensity of 350 μmol m^−2^s^−1^), day/night temperatures of 22 °C/18 °C, and relative humidity of 70 ± 10%. At 3 days post-inoculation, the seedlings were observed and photographed using a Nikon D700 camera, then the roots were harvested for qPCR analysis. The relative accumulation of *P. sojae* in roots was measured based on the relative expression of *P. sojae* housekeeping gene *PsACT* (GenBank accession no. XM_009530461.1) to soybean housekeeping gene (Glyma.18G290800.1) (ΔCt = Ct*_HK_*_of *P. sojae*_ *−* Ct*_HK_*_of soybean_).

### 5.6. Yeast-One-Hybrid Assay

The CDS sequence of *GmARF2, GmARF11,* and *GmARF15* was amplified and inserted into the pGADT7 prey vector. The specific DNA fragment P (−461 to −416 region of the *GmPT10d* promoter) was synthesized and recombined into pHIS2 vector. Co-transformation of the fusion plasmids pGADT7-GmARF2/GmARF11/GmARF15 and pHIS2-P into strain Y187 cells using the PEG/LiAC method, according to the protocol handbook (Clontech, Mountain View, CA, USA). Then, the transformed yeast colonies were tested and cultured on SD/-Leu-Trp medium for 3 days at 30 °C. Subsequently, each yeast colony was washed off with water, spotted on SD/-His-Leu-Trp medium supplemented with 100 mM 3-aminotriazole, and cultured for 3 days at 30 °C. The interaction was judged by the growth of yeast on the selection medium.

### 5.7. Electrophoretic Mobility Shift Assay

The CDS sequence of *GmARF15* was amplified and inserted into pGEX-4T-1 vector (GE Amersham, Shanghai, China) for the production of GmARF15-GST protein. The recombinant fusion plasmid was transformed into *Escherichia coli* BL21 (DE3) cells. GmARF15-GST production was induced with 0.5 mM isopropyl-β-D-thiogalactoside and carried out at 37 °C for 5 h. Subsequently, the DNA binding activity of GmARF15 was examined using digoxigenin-ddUTP-labeled double-stranded oligonucleotide P (−461 to −416 region of the *GmPT10d* promoter) probe. EMSA was performed following the protocol outlined by Hou et al. (2019) [54]. The signal was detected using an Odyssey CLx infrared fluorescence imaging system (LI-COR, Lincoln, Nebraska, USA) [55].

### 5.8. Subcellular Localization

The CDS sequence of *GmARF15* was cloned into the pCAMBIA1302 expression vector, which was fused to the GFP reporter gene. Then, the recombinant vector pCAMBIA1302-*GmARF15* and the empty vector pCAMBIA1302 were respectively transformed into *Agrobacterium* GV3101 competent cells and transiently expressed in the leaves of *Nicotiana benthamiana*. Three days later, fluorescence images were captured using a TCS SP2 confocal spectral microscope (Leica, Wetzlar, Germany).

### 5.9. Transactivation Assay

The CDS sequence of *GmARF15* was cloned into pCAMBIA3301 to generate the effector plasmid. The AuxRE TGTCTC was multimerized three times and inserted upstream of the CaMV35S promoter (−42 to +8) containing a TATA box, which was inserted into pXGUS-P and fused to the *GUS* gene to generate the reporter plasmid. The effector plasmid (35S::*GmARF15*) and the reporter plasmid (35S mini::*GUS*, 3 × TTTTTT-35S mini::*GUS* or 3 × TGTCTC-35S mini::*GUS*) were co-transformed into Arabidopsis protoplasts as described by Yoo et al. (2007) [56]. The transfected cells were incubated at 22 °C in light for 18–20 h. GUS activity was determined following the methods described by Lu et al. (1998) [57].

### 5.10. Transient Luciferase Assay

The transient LUC assay was performed as previously described [58]. The luciferase gene *LUC* was inserted into the pBI121 vector to replace *GUS* as the reporter gene. Subsequently, the 1.5 kb region upstream of the *GmPT10d* was cloned into the modified pBI121 vector and fused to the *LUC* reporter gene. The effector plasmid 35S::*GmARF15* and the reporter plasmid p*GmPT10d*::*LUC* were co-transformed into *Agrobacterium* GV3101competent cells and transiently expressed in tobacco leaves. The tobacco leaves transformed with p*GmPT10d*::*LUC* were used as control. After 3 days of incubation, luciferase activity was measured using the Dual-Luciferase Reporter Assay System Kit (Promega, Madison, WI, USA).

### 5.11. SA Content Evaluation

Endogenous SA was extracted from the roots of soybean seedlings at 24, 48, and 72 h post-inoculation. The SA content was measured using a SA ELISA kit (Meimian, Jiangsu, China). Measurement was carried out following the manufacturer’s protocol. SA content was measured using a fluorescence detector (excitation at 305 nm and emission at 405 nm) on a Waters 515 system (Waters, Milford, MA, USA).

### 5.12. Statistical Analysis

All statistical methods are annotated in the figure captions. Three biological replicates were used for each sample and Student’s *t*-test (* *p* < 0.05, ** *p* < 0.01) was used to determine statistical significance. Error bars represent ±SD.

### 5.13. Primer Sequences Used in the Present Study

The specific primers used for all assays are listed in Table S1.

## Figures and Tables

**Table 1:** Elements in the 1.5 kb promoter sequence of *GmARF15*.

Site Name	Sequence	Function	Position
3-AF1 binding site	TAAGAGAGGAA	light responsive element	−1274
AP-1	TGAGTTAG		718
AT~TATA-box	TATATA		+384, +386
Box 4	ATTAAT	part of a conserved DNA module involved in light responsiveness	+359, −874
CAAT-box	CAAT, CAAAT	common cis-acting element in promoter and enhancer regions	+44, −60, −72, −99, −320, +357, −569, +590, +663, +687, −893, −1009, +1019, −1248, +1386, +1453, +1483, +57, −134, +472, −490, −552, −778, +853, −941, −1413
CAT-box	GCCACT	cis-acting regulatory element related to meristem expression	−729, +733
GA-motif	ATAGATAA	part of a light responsive element	+897
MYB	CAACCA		−555, +821, +1444
MYB-like sequence	TAACCA		+817, −1477
MYC	CATTTG		+133, +940
Myb	TAACTG		−1316
TATA-box	TATA, TATTTAAA, ATATAA		+87, −857, −840, +170, −889, −1241, +374, +164, −1096, +388, −831, +807, −220, −912, +373, +387
TC-rich repeats	GTTTTCTTAC	cis-acting element involved in defense and stress responsiveness	+250, +1161
TCA	TCATCTTCAT		+1348, +1351
TCA-element	CCATCTTTTT	cis-acting element involved in salicylic acid responsiveness	+824, −711
TCT-motif	TCTTAC	part of a light responsive element	+848, +1165
Unnamed__1	CGTGG		−1119
Unnamed__4	CTCC		+83, −1123, −657, −1120, +424, −1199, +1116
WUN-motif	TAATTACTC		+36

## Data Availability

Data are contained within the article and Supplementary Materials.

## Supplementary Materials

The following supporting information can be downloaded at: https://www.mdpi.com/article/10.3390/ijms26010191/s1.
