# Supplementary figures and images for: GmARF15 Enhances the Resistance of Soybean to Phytophthora sojae by Promoting GmPT10d Expression in Response to Salicylic Acid Signalling
Source: Int J Mol Sci. 2024 Dec 29;26(1):191. doi: 10.3390/ijms26010191 (PMC11720048; doi:10.3390/ijms26010191)

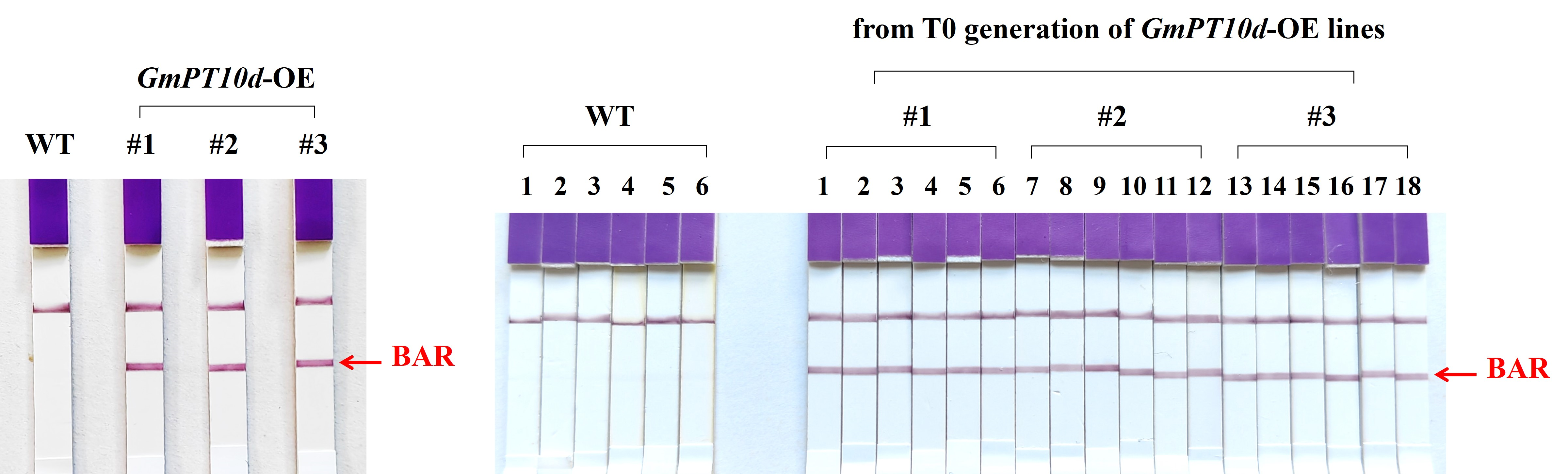

Supplement: Supplementary file 1 [file ijms-26-00191-s001.zip › Figure S1.tif]

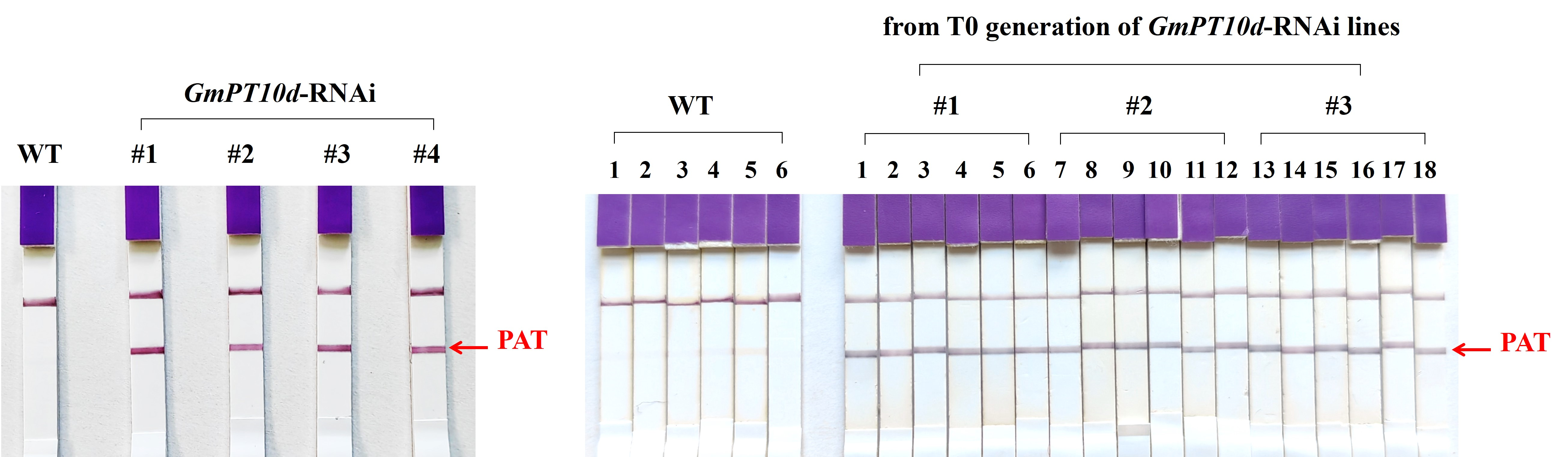

Supplement: Supplementary file 1 [file ijms-26-00191-s001.zip › Figure S2.tif]

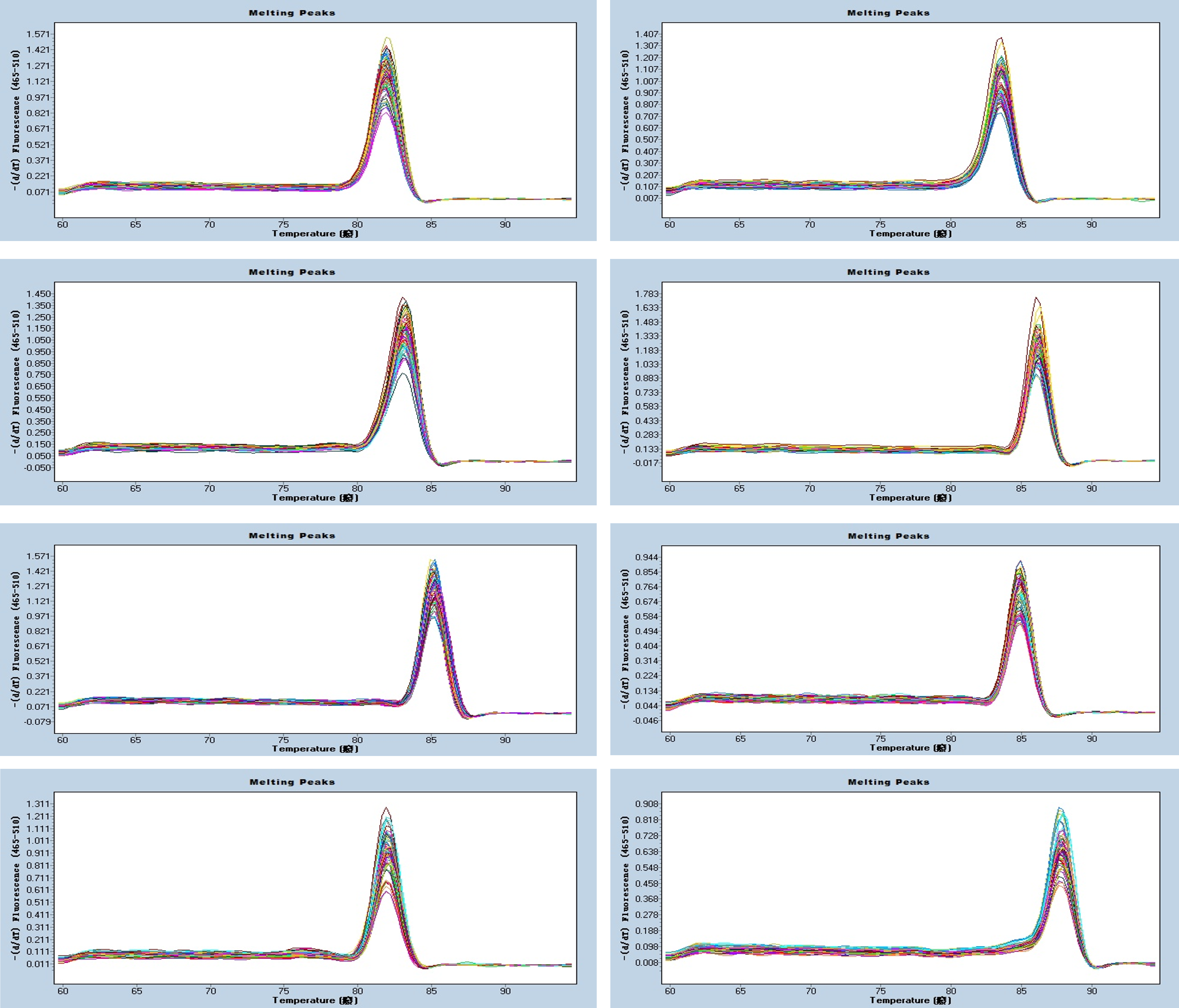

Supplement: Supplementary file 1 [file ijms-26-00191-s001.zip › Figure S3.tif]

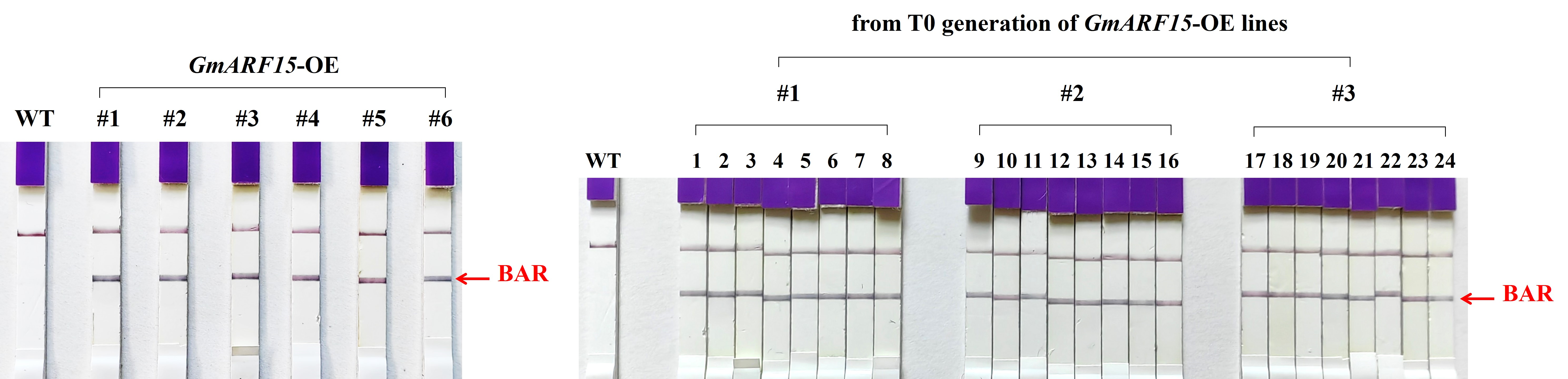

Supplement: Supplementary file 1 [file ijms-26-00191-s001.zip › Figure S4.tif]
